# A comparative analysis of syntactic complexity in argumentative essays from rhetorical perspective: ChatGPT vs. English native speakers

**DOI:** 10.1371/journal.pone.0329410

**Published:** 2025-08-01

**Authors:** Wenlong Liu, Xianming Liu

**Affiliations:** School of Foreign Languages and Cultures, Jilin University, Changchun, China; STL UMR8163 CNRS, FRANCE

## Abstract

This study investigates the syntactic complexity of argumentative essays generated by ChatGPT in comparison to those written by native speakers. By examining cross-rhetorical-stage variation in syntactic complexity, we explore how ChatGPT’s writing aligns with or diverges from human argumentative writing. The results reveal that ChatGPT and native speakers exhibit similar patterns in mean length of sentence in the thesis stage, mean length of T-unit and complex nominals per T-unit in the conclusion stage. However, ChatGPT showed a preference for coordination structures across all stages, relying more on parallel constructions, and native speakers used subordination structure and verb phrases more frequently across all stages. Additionally, ChatGPT’s syntactic complexity was characterized by lower variability across multiple measures, indicating a more uniform and formulaic output. These findings underscore the differences between ChatGPT and native speakers in syntactic complexity and rhetorical functions in argumentative essays, therefore contributing to our understanding of ChatGPT’s argumentative writing performance and providing valuable insights for ChatGPT integration into writing instruction.

## Introduction

In recent decades, scholars have increasingly explored how syntactic complexity interacts with rhetorical units [1–3]. Previous studies in this field have primarily focused on syntactic complexity in research articles [2,3], investigating how syntactic complexity varies across rhetorical moves and how it is influenced by factors such as academic disciplines [4] and writer groups [5]. While these studies have enhanced our comprehension of syntactic complexity in relation to rhetorical moves, the focus on genre-specific research articles limits their broader applicability, leaving other forms of writing relatively understudied [6].

Argumentative writing is universally acknowledged as a crucial component of literacy, particularly essential for undergraduates to fulfill their academic requirements [7,8]. Through composing argumentative essays, students are expected to analyze complex issues, evaluate evidence, and display critical thinking abilities [9]. Despite the significance of argumentative essays, student writing often reveals a lack of “audience awareness” [10], which required the use of appropriate rhetorical techniques to engage targeted readers. appropriate rhetorical techniques to engage targeted readers. In addition to rhetorical competence, syntactic complexity, another key aspect in student writing [11], also warrants attention, particularly in terms of how it interacts with rhetorical structure in argumentative essays. Yet this area remains underexplored, calling for further investigation.

To support the integration of syntactic complexity and rhetorical structures in argumentative writing, an increasing number of AI tools are now available to help. One such tool is ChatGPT. Created by OpenAI, ChatGPT stands out as a sophisticated chatbot capable of generating human-like text based on input prompt. Its functions in argumentative writing have been validated in terms of the capability to produce natural language that addresses dialogical, structural, and linguistic challenges [12]. For instance, ChatGPT can generate accurate argumentative summaries on provided topic based on the prompts of contexts and targeted audience [13]. However, introducing ChatGPT into writing instruction is premised on more accurate grasp of the writing performance and characteristics of ChatGPT and human writers. Given that native speaker texts are often regarded as authentic models of language use and serve as a model for language learners [14], such comparison enables us to assess whether, and in what aspects, ChatGPT can function as an effective model for student writing. It is therefore important to examine the extent to which ChatGPT-generated texts resemble or diverge from native speaker writing.

Our study aims to examine the syntactic complexity of specific rhetorical stages and how it varies across these stages in ChatGPT-generated argumentative texts through a comparative analysis with texts written by native speakers. By analyzing texts of similar length and same topic, this study not only provides empirical evidence for identifying ChatGPT-generated texts, but also offers pedagogical implications for using ChatGPT as a writing assistant. Specifically, the study addresses the following research questions:

1. How does the syntactic complexity differ between argumentative essays generated by ChatGPT and those written by native speakers across rhetorical stages?
2. If syntactic complexity varies within each group across rhetorical stages, how do ChatGPT and native speakers exhibit these different patterns of variation?

Based on previous findings on human and AI-generated texts [15,16], the study hypothesizes that:

H1: ChatGPT-generated argumentative texts differ significantly from those written by native speakers in syntactic complexity across rhetorical stages.

H2: The pattern of variation in syntactic complexity across rhetorical stages differs between ChatGPT and native speakers.

## Literature review

This section reviews literature on ChatGPT and writing, rhetorical moves in argumentative writing, and the relationship between syntactic complexity and rhetorical moves, outlining previous findings and research gaps relevant to the current study.

### ChatGPT and writing

ChatGPT has garnered significant attention from scholars across various domains since its release, sparking numerous studies and inquiries into its capabilities and implications. Built on advanced machine learning algorithms and extensive datasets, ChatGPT is capable of dealing with a wide range of tasks across different prompts, such as text generation, modification and translation [17]. But among its various applications, writing is considered the most useful function [18]. Recent studies have shown that ChatGPT can produce human-like texts [19], assisting students in improving grammar, coherence, and stylistic refinement in their writing. However, whether it can produce texts with coherent rhetorical structure remains less explored [20].

Beyond these contributions, attempts to investigate differences between ChatGPT-generated and human-written texts also lay a foundation for exploring ChatGPT’s potential in writing. Tudino and Qin noted that, compared with human-authored texts, ChatGPT-generated research articles tended to overuse low-frequency “academic” vocabulary, underutilize subordination, and display less syntactic and semantic variation [21]. Jiang and Hyland [15,16] analyzed argumentative essays and found ChatGPT texts included fewer lexical bundles and engagement markers, reflecting a more rigid and formulaic style with limited capacity for constructing interactional arguments. Conversely, native-speaker essays exhibited a stronger authorial presence and made greater use of engagement markers, indicating more interactive and persuasive discourse. These findings collectively underscore the distinct linguistic strategies employed in ChatGPT-generated and human-authored texts while also revealing ChatGPT’s limitations in argumentative writing.

In addition to aforementioned features relevant to argumentative writing, rhetorical moves, functioning as discoursal units that help achieve coherent communicative goals, are also essential in structuring argumentative essays [22]. But to date, only a few scholars have focused on the rhetorical moves of ChatGPT-generated texts. Kong and Liu [20], for instance, found both human-written and ChatGPT-generated abstracts frequently included the moves of presenting the research and describing the methodology. Nevertheless, unlike ChatGPT, human-written abstracts often integrated the methodology move with the results move and exhibited a different overall sequence of rhetorical moves. While this research has provided new insights, it remains largely limited to abstracts, leaving other important genres like argumentative essays underexplored. Given the importance and fundamental role of argumentative writing in academic contexts, this lack of attention to rhetorical structure in ChatGPT-generated argumentative essays reveals an important research gap. A comprehensive understanding of the rhetorical structures in ChatGPT-generated and human-written argumentative essays is essential for improving ChatGPT’s ability to meet the specific demands of this genre and refining its role as a writing assistant in academic contexts.

### Syntactic complexity and rhetorical moves in argumentative writing

Syntactic complexity, referring to the diversity, sophistication, and elaboration of syntactic structures in language production [11,23], is widely recognized as a key indicator of linguistic proficiency [24]. Research has indicated that higher language proficiency is often related to more complex syntactic structures [25, 26, 27]. In addition, employing appropriate syntactic complexity to address different registers, genres, and tasks is also a sign of mature writers [28–31]. For instance, argumentative essays typically demonstrate greater syntactic complexity than narrative essays, particularly in terms of the length of production units, coordination, and phrasal sophistication [31], reflecting specific linguistic demands of different genres.

Therefore, to complete genre-based writing, rhetorical moves, which guide readers to the theme and substantive content of a text [32,33], also deserve our attention. Rhetorical moves refer to functional units, serving specific communicative purposes that collectively support the text’s overall rhetorical goal within a particular social context [3]. These moves vary across genres and sections of academic texts, reflecting their unique rhetorical goals. For example, Hyland’s [34] model for argumentative essays includes three stages, namely Thesis, Argument, and Conclusion, which correspond to rhetorical moves as defined, each fulfilling specific communicative functions to persuade readers of the central statement’s correctness [6].

In recent years, increasing scholarly attention has been directed toward the relationship between syntactic complexity and rhetorical moves in argumentative writing [3,4]. Much of this research has focused on research articles, particularly in sections such as abstracts and introductions [4,35]. However, limited studies have examined this relationship in argumentative essays. To our knowledge, only Zhang and Cui [6] revealed significant syntactic differences across rhetorical stages in native and L2 learners’ argumentative writing.

While all of the above studies focus on comparisons of language produced by humans, there is insufficient research comparing AI-generated language with regard to its syntactic complexity and rhetorical moves. Zindela [36] compared a single syntactic complexity measure between ChatGPT-generated and L2 human-written argumentative essays, finding that L2 learners’ essays exhibited lower mean length of sentence than those written by ChatGPT. However, studies comparing ChatGPT-generated texts with those of human like native speakers, particularly with a focus on syntactic complexity across rhetorical moves, are notably absent. By examining these differences, educators and researchers can also develop a more profound insight into how to effectively incorporate ChatGPT into writing instruction with academic standards. Therefore, considering the literature and research gaps above, this study examines the syntactic complexity of ChatGPT-generated essays compared with those written by native speakers, focusing on how these features vary across rhetorical stages and their implications for argumentative effectiveness.

## Method

This section outlines the methodology employed to compare syntactic complexity in ChatGPT-generated and native speaker argumentative essays. It is organized into four subsections. The first describes the data sources and selection criteria for both corpora, ensuring consistency in topic and length. The second details the procedures for annotating rhetorical stages based on Hyland’s framework. The third introduces the syntactic complexity measures adopted from Lu’s L2 Syntactic Complexity Analyzer (L2SCA). The last explains the statistical methods used to analyze group differences in syntactic complexity across rhetorical stages.

### Data

A native speaker corpus, sourced from the International Corpus Network of Asian Learners of English, consists of 73 essays by American students across Social Sciences, Sciences and Technology, Humanities, and Life Sciences [37]. Among the native speaker section of ICNALE, 57% of the participants are identified as American. However, only 73 essays were confirmed to be written by American students, as the remaining participants were marked as “N/A” or categorized as English teachers or adults with varied job backgrounds. To ensure demographic consistency and maintain the validity of the comparison, this subset of 73 essays was selected for analysis.

To ensure the reliability of this analysis, several key factors were carefully controlled. First, all essays were produced under standardized conditions, including a time limit and a word count range of 200–300 words. Second, to minimize potential linguistic variation caused by differences in essay topics, this study selected one of ICNALE’s two common argumentative topics, “Smoking should be completely banned at all restaurants in the country,” ensuring topic consistency across the dataset. This controlled design enhances the comparability of essays generated by ChatGPT and native speakers, enabling a targeted analysis of syntactic complexity and rhetorical patterns.

The ChatGPT corpus was created by prompting the GPT-4o mini model to simulate argumentative writing typical of competent university students, chosen for its feasibility in this study and its current availability as a free tool for public use. The data collection process utilized a standardized prompt, adapted from Jiang and Hyland’s [15] design, instructing the model as follows: “You are a competent university-student writer of English tests for academic purposes. Write an argumentative essay on the topic ‘Smoking should be completely banned at all restaurants in the country.’ You can choose your stance and fulfill the argumentation without giving the title. The essay is at least 200 words but no more than 300 words.” Due to inherent word count constraints in ChatGPT, essays could only be generated one at a time, requiring the prompt to be repeated 73 times to produce the full dataset. This process ensured consistency in task requirements across both corpora. The resulting essays varied in stance (pro and con) and rhetorical strategies, providing a robust basis for comparing the syntactic complexity and rhetorical stages of ChatGPT-generated texts with those written by native speakers.

After collecting a total of 146 argumentative essays, all texts were manually converted into Word files with formatting consistency and no typos or grammatical errors being rectified. The essays from the two groups were organized into separate datasets to facilitate systematic comparison. Finally, two files were generated, comprising 19,868 words for the ChatGPT’s argumentative essays and 15,574 words for native speakers’ argumentative essays respectively. The average word count per ChatGPT’s argumentative essays is 272.164 words, while the average word count per native speakers’ argumentative essays is 213.342 words, as detailed in Table 1.

**Table 1:** Data overview.

Group	N	T	M	SD
GPT	73	19,868	272.164	20.675
NS	73	15,574	213.342	9.741
All	146	35,442	242.753	33.621

Note: GPT = ChatGPT, NS = Native Speakers, N = The Number of Sample, T = Total Word Count; M = Average Word Length; SD = Standard Deviation of Word Length.

### Rhetorical stage annotation

The texts were manually annotated for rhetorical stages based on Hyland’s [34] three-stage framework in argumentative essays, which is shown in Table 2. First, two experienced Chinese university professors, both possessing more than 10 years of expertise in teaching English writing, were invited to independently annotate a sample of 20 essays (10 written by native speakers and 10 generated by ChatGPT). To minimize potential bias, the annotators were not informed of the source of each essay during the annotation process. After completing their initial annotations, the inter-rater reliability was calculated using Cohen’s kappa coefficient, yielding a value of 0.773, which indicates substantial agreement. The two teachers then compared results and discussed all instances where they disagreed on the boundaries or labels of rhetorical stages, a common challenge that other researchers have also encountered in manual annotation [38]. Discrepancies typically involved ambiguous transitions between moves, such as between argument development and conclusion. These were resolved through collaborative discussion and reference to Hyland’s framework until consensus was reached. After that, the remaining 126 essays were independently annotated by the two teachers. Finally, a thorough review of all annotations was conducted to ensure consistency. The inter-rater reliability was calculated using Cohen’s kappa coefficient, yielding a value of 0.898, which indicates almost prefect agreement. During this review, the annotations were cross-checked again for adherence to Hyland’s framework, focusing on the accuracy of rhetorical stage labels, completeness of rhetorical structure, and consistency in boundary demarcation. Any remaining ambiguities were resolved through discussion.

**Table 2:** Rhetorical stage framework for argumentative essays by ChatGPT and native speakers.

Stage	Description	Proportion %		
		GPT (N = 73)	NS (N = 73)	Total (N = 146)
Thesis (S1)	Introducing the proposition to be argued.	100.000	82.192	91.096
Argument (S2)	Discussing grounds for thesis.	100.000	100.000	100.000
Conclusion (S3)	Synthesizing discussion and affirms the validity of the thesis.	100.000	79.452	89.726

Note: GPT = ChatGPT, NS = Native Speakers, N = The Number of Sample.

### Syntactic complexity measurement

Syntactic complexity refers to the degree of variation, elaboration, and sophistication in language production, and is commonly conceptualized as a multidimensional construct involving global complexity, subordination, coordination, clausal elaboration, and phrasal complexity [39]. To evaluate the syntactic complexity of each argumentative essay, we utilized the L2 Syntactic Complexity Analyzer (L2SCA), a software tool created by Lu Xiaofei. [40]. This is because this tool closely aligns with the definition of syntactic complexity, as it operationalizes the construct across five dimensions through 14 measures, which were specially selected from over 100 measures reviewed by Wolfe-Quintero et al. [41] and Ortega [42]. Moreover, L2SCA has demonstrated reliable accuracy in computing complexity scores [40] and has been widely applied in studies on syntactic complexity and rhetorical moves. Following prior studies that employed L2SCA to investigate syntactic complexity from a rhetorical perspective [4,23], all 14 measures were included in our analysis. Table 3 presents a detailed review of all measures, including their corresponding codes and definitions.

**Table 3:** Syntactic complexity measures from L2SCA [40].

Measure	Code	Definition
Length of production unit		
Mean length of clause	MLC	# of words/# of clauses
Mean length of sentence	MLS	# of words/# of sentences
Mean length of T-unit	MLT	# of words/# of T-units
Amount of subordination		
Clauses per T-unit	C/T	# of clauses/# of T-units
Complex T-units per T-unit	CT/T	#of complex T-units/# of T-units
Dependent clauses per clause	DC/C	#of dependent clauses/# of clauses
Dependent clauses per T-unit	DC/T	#of dependent clauses/# of T-units
Amount of coordination		
Coordinate phrases per clause	CP/C	# of coordinate phrases/# of clauses
Coordinate phrases per T-unit	CP/T	# of coordinate phrases/# of T-units
T-units per sentence	T/S	# of T-units/# of sentences
Degree of phrasal sophistication		
Complex nominals per clause	CN/C	# of complex nominals/# of clauses
Complex nominals per T-unit	CN/T	# of complex nominals/# of T-units
Verb phrases per T-unit	VP/T	# of verb phrases/# of T-units
Overall sentence complexity		
Clauses per sentence	C/S	# of clauses/# of sentences

To assess syntactic complexity based on stages, we created individual plain text files for each ChatGPT’s and native speaker’s argumentative essays. These files were named S1GPT1, S1NS1, S2GPT1 and S2NS1, etc. Then, the L2SCA tool was employed to calculate the values of the 14 measures representing syntactic complexity in these files.

### The statistical analysis

The statistical analysis was conducted using SPSS 27.0. First, the test for normality and homogeneity of variance was carried out to evaluate the data distribution, indicating that both ChatGPT’s and native speakers’ essays were normally distributed across all syntactic complexity measures. However, some indices failed to meet the assumption of homogeneity of variance. Thus, the Mann-Whitney U test, a non-parametric counterpart to the t-test, assessed differences across 14 syntactic complexity measures in both groups.

## Results

This section presents the results on syntactic complexity and rhetorical stages in argumentative essays produced by ChatGPT and by native English speakers. It is organized into two subsections. The first compares syntactic complexity between ChatGPT-generated and native-speaker essays across rhetorical stages, identifying significant differences in various syntactic measures. The second explores how syntactic complexity varies across rhetorical stages within each group, with patterns illustrated through statistical analysis and figures to show distinct trends in the writing of ChatGPT and native speakers.

### The difference between ChatGPT and native speakers by rhetorical stages

Table 4 shows the difference of syntactic complexity measures between ChatGPT and native speakers across rhetorical stages. Although no significant differences were observed in MLS in the thesis stage or in MLT and CN/T in the conclusion stage in both groups, most other measures showed significant group differences. ChatGPT demonstrated significantly higher values than native speakers in some syntactic complexity measures across rhetorical stages. MLC was significantly higher for ChatGPT in the thesis (15.259 vs. 8.596, p < .001), argument (12.349 vs. 8.726, p < .001), and conclusion stages (19.608 vs. 10.087, p < .001). Similarly, ChatGPT demonstrated significantly higher values in CN/C across all stages, including the thesis (2.154 vs. 1.043, p < .001), argument (1.787 vs. 0.910, p < .001), and conclusion stages (2.605 vs. 1.206, p < .001). CP/C was also significantly more frequent in ChatGPT’s writing at every stage, with CP/C in the thesis (0.478 vs. 0.069, p < .001), argument (0.439 vs. 0.145, p < .001) and conclusion stages (0.780 vs. 0.154, p < .001). CP/T followed a similar pattern, with ChatGPT showing significantly higher values in the thesis (0.602 vs. 0.141, p < .001), argument (0.622 vs. 0.328, p < .001) and conclusion stages (0.824 vs. 0.355, p < .001). MLT was significantly higher for ChatGPT in the thesis stage (19.361 vs. 17.777, p = .021), while CN/T were significantly higher for ChatGPT in the thesis (2.726 vs. 2.218, p < .001) and argument stages (2.538 vs. 2.136, < .001).

**Table 4:** The difference of syntactic complexity between ChatGPT and native speakers across rhetorical stages.

Index	Thesis			Argument			Conclusion		
	GPT	NS	p	GPT	NS	p	GPT	NS	p
MLC	15.259 (5.628)	8.596 (2.804)	<.001^***^	12.349 (1.414)	8.726 (1.394)	<.001^***^	19.608 (5.305)	10.087 (4.026)	<.001^***^
MLS	20.847 (4.836)	21.944 (9.374)	.867	18.218 (1.714)	28.805 (7.011)	<.001^***^	21.747 (4.040)	26.805 (10.465)	<.001^***^
MLT	19.361 (5.273)	17.777 (7.396)	.021^*^	17.512 (1.777)	20.528 (5.410)	<.001^***^	20.795 (4.397)	21.439 (10.331)	.092
C/T	1.363 (0.460)	2.186 (0.943)	<.001^***^	1.431 (0.183)	2.368 (0.563)	<.001^***^	1.100 (0.243)	2.310 (1.215)	<.001^***^
CT/T	0.330 (0.371)	0.747 (0.363)	<.001^***^	0.364 (0.142)	0.780 (0.192)	<.001^***^	0.135 (0.248)	0.715 (0.396)	<.001^***^
DC/C	0.201 (0.216)	0.448 (0.228)	<.001^***^	0.292 (0.083)	0.509 (0.102)	<.001^***^	0.087 (0.151)	0.422 (0.233)	<.001^***^
DC/T	0.368 (0.461)	1.161 (0.924)	<.001^***^	0.433 (0.180)	1.246 (0.490)	<.001^***^	0.126 (0.227)	1.173 (1.022)	<.001^***^
CP/C	0.478 (0.311)	0.069 (0.172)	<.001^***^	0.439 (0.133)	0.145 (0.112)	<.001^***^	0.780 (0.353)	0.154 (0.318)	<.001^***^
CP/T	0.602 (0.373)	0.141 (0.353)	<.001^***^	0.622 (0.176)	0.328 (0.242)	<.001^***^	0.824 (0.352)	0.355 (0.603)	<.001^***^
T/S	1.100 (0.206)	1.261 (0.405)	.013^*^	1.043 (0.067)	1.432 (0.252)	<.001^***^	1.062 (0.166)	1.348 (0.457)	<.001^***^
CN/C	2.154 (0.862)	1.043 (0.506)	<.001^***^	1.787 (0.285)	0.910 (0.258)	<.001^***^	2.605 (0.940)	1.206 (0.775)	<.001^***^
CN/T	2.726 (0.856)	2.218 (1.569)	<.001^***^	2.538 (0.410)	2.136 (0.747)	<.001^***^	2.772 (0.911)	2.589 (1.707)	.087
VP/T	1.680 (0.634)	2.773 (1.445)	<.001^***^	2.143 (0.261)	3.277 (0.844)	<.001^***^	2.000 (0.511)	3.505 (1.768)	<.001^***^
C/S	1.479 (0.473)	2.703 (1.221)	<.001^***^	1.490 (0.192)	3.347 (0.838)	<.001^***^	1.174 (0.344)	2.963 (1.460)	<.001^***^

Note: *p < .05, ***p < .001. Each cell contains two numbers, where the first indicates the mean value and the second, presented in parentheses, represents the standard deviation. GPT and NS denote ChaGPT and native speakers.

To compare, native speakers demonstrated significantly higher values than ChatGPT in many other syntactic complexity measures. In all the measures related to amount of subordination, native speakers showed significantly higher values. For C/T, native speakers exhibited higher values in the thesis (2.186 vs. 1.363, p < .001), argument (2.368 vs. 1.431, p < .001), and conclusion stages (2.310 vs. 1.100, p < .001). Similarly, native speakers’ texts suggested significantly higher CT/T in the thesis (0.747 vs. 0.330, p < .001), argument (0.780 vs. 0.364, p < .001), and conclusion stages (0.715 vs. 0.135, p < .001). DC/C followed the same pattern, with native speakers showing significantly higher values in the thesis (0.448 vs. 0.201, p < .001), argument (0.509 vs. 0.292, p < .001), and conclusion stages (0.422 vs. 0.087, p < .001). DC/T was also significantly higher for native speakers across all stages including thesis (1.161 vs. 0.368, p < .001), argument (1.246 vs. 0.433, p < .001), and conclusion stages (1.173 vs. 0.126, p < .001). Native speakers also demonstrated significantly higher values than ChatGPT in measures of T/S, VP/T and C/S across all stages. For T/S, native speakers showed significantly higher values in the thesis (1.261 vs. 1.100, p = .013), argument (1.432 vs. 1.043, p < .001), and conclusion stages (1.348 vs. 1.062, p < .001). Similarly, VP/T was significantly higher for native speakers in the thesis (2.773 vs. 1.680, p < .001), argument (3.277 vs. 2.143, p < .001), and conclusion stages (3.505 vs. 2.000, p < .001). For C/S, native speakers demonstrated significantly higher values in the thesis (2.703 vs. 1.479, p < .001), argument (3.347 vs. 1.490, p < .001), and conclusion stages (2.963 vs. 1.174, p < .001). Beyond these measures, native speakers produced significantly longer sentences and T-units in certain stages. MLS was significantly higher for native speakers in the argument (28.805 vs. 18.218, p < .001) and conclusion stages (26.805 vs. 21.747, p < .001), while MLT was significantly higher in the argument stage (20.528 vs. 17.512, p < .001). In addition to mean differences, the two groups also differed in terms of variation. Standard deviations for native speakers were higher than those for ChatGPT in many syntactic complexity measures, except for MLC, CP/C, and CN/C across all stages, and CP/T in the thesis stage.

### Patterns of syntactic complexity variation across rhetorical stages for ChatGPT and native speakers

Table 5 presents the stage-specific differences in syntactic complexity between ChatGPT and native speakers. Combined with findings from Table 4, for ChatGPT, significant differences were found across rhetorical stages in most syntactic complexity measures, except CN/T. For example, T/S was higher in the thesis stage than other two stages. For native speakers, differences were observed across rhetorical stages in nine syntactic complexity measures, except CT/T, DC/C, DC/T, CN/C and CN/T. For instance, MLS, C/T, T/S and C/S show the highest value in the argument stage.

**Table 5:** Stage-specific differences in syntactic complexity between ChatGPT and native speakers.

Index		H	p	Post-hoc comparisons		
				S1-S2.	S2-S3.	S1-S3.
MLC	GPT	77.012	<.001^***^	.002^**^	.000^***^	<.001^***^
	NS	8.002	.018^*^	.081	.225	.005^**^
MLS	GPT	37.374	<.001^***^	<.001^***^	<.001^***^	.113
	NS	22.501	<.001^***^	<.001^***^	.126	.003^**^
MLT	GPT	24.500	<.001^***^	.052	<.001^***^	.009^**^
	NS	10.315	.006^**^	.001^***^	.297	.044^*^
C/T	GPT	55.165	<.001^***^	<.001^***^	<.001^***^	<.001^***^
	NS	7.263	.026^*^	.018^*^	.026^*^	.910
CT/T	GPT	36.675	<.001^***^	.023^*^	<.001^***^	<.001^***^
	NS	1.742	.419	/	/	/
DC/C	GPT	47.812	<.001^***^	<.001^***^	<.001^***^	<.001^***^
	NS	4.291	.117	/	/	/
DC/T	GPT	50.027	<.001^***^	<.001^***^	<.001^***^	<.001^***^
	NS	4.999	.082	/	/	/
CP/C	GPT	43.958	<.001^***^	.554	<.001^***^	<.001^***^
	NS	31.942	<.001^***^	<.001^***^	.001^**^	.026^*^
CP/T	GPT	20.020	<.001^***^	.675	<.001^***^	<.001^***^
	NS	34.029	<.001^***^	<.001^***^	.002^**^	.012^*^
T/S	GPT	6.842	.033^*^	.225	.009^**^	.161
	NS	15.534	<.001^***^	<.001^***^	.015^*^	.182
CN/C	GPT	32.281	<.001^***^	.029	<.001^***^	<.001^***^
	NS	4.833	.089	/	/	/
CN/T	GPT	3.832	.147	/	/	/
	NS	2.140	.343	/	/	/
VP/T	GPT	38.398	<.001^***^	<.001^***^	.047^*^	<.001^***^
	NS	12.434	.002^**^	<.001^***^	.372	.016^*^
C/S	GPT	40.475	<.001^***^	.146	<.001^***^	<.001^***^
	NS	13.231	<.001^***^	<.001^***^	.017^*^	.293

Note: *p < .05, **p < .01,***p < .001. The H statistic quantifies the variance in rank distributions across groups; a higher H value indicates greater differences in median ranks across the groups being compared. A slash (/) denotes no significant difference between writing groups. S1, S2 and S3 represent Thesis, Argument and Conclusion. GPT and NS denote ChaGPT and native speakers.

**Fig 1:**
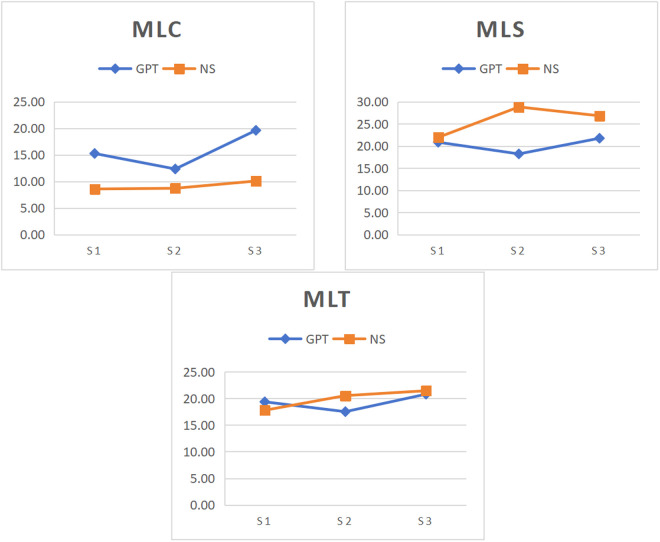
Variation of length of production unit across rhetorical stages in GPT and NS. Note: S1, S2 and S3 represent Thesis, Argument and Conclusion. GPT and NS denote ChaGPT and native speakers.

**Fig 2:**
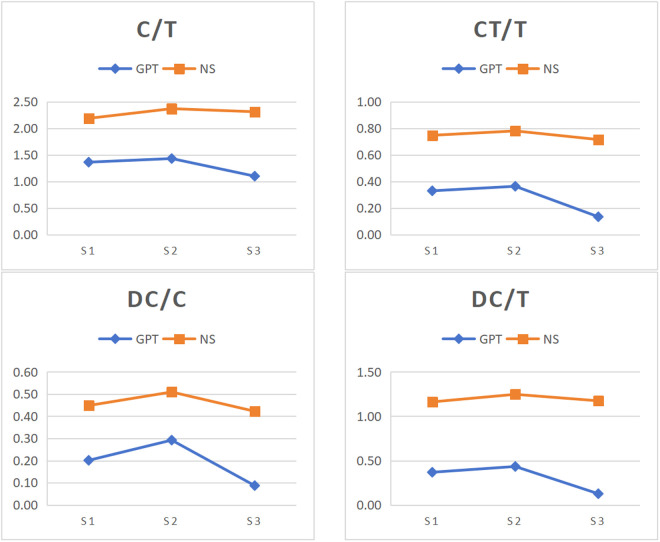
Variation of amount of subordination across rhetorical stages in GPT and NS. Note: S1, S2 and S3 refer to Thesis, Argument and Conclusion. GPT and NS denote ChaGPT and native speakers.

**Fig 3:**
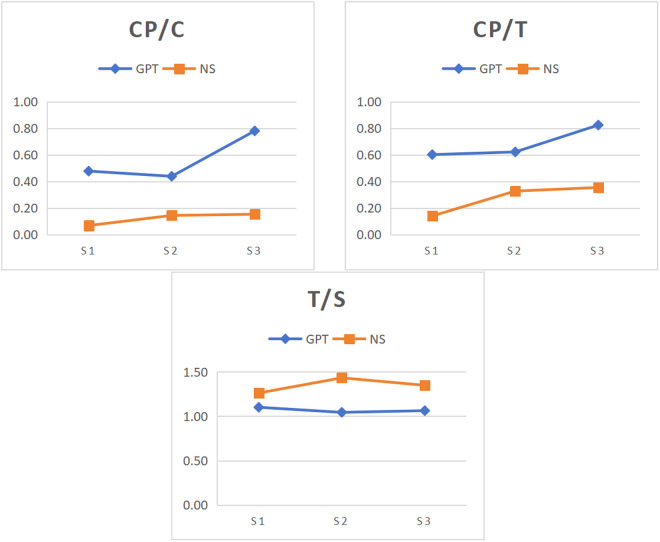
Variation of amount of coordination across rhetorical stages in GPT and NS. Note: S1, S2 and S3 represent Thesis, Argument and Conclusion. GPT and NS denote ChaGPT and native speakers.

**Fig 4:**
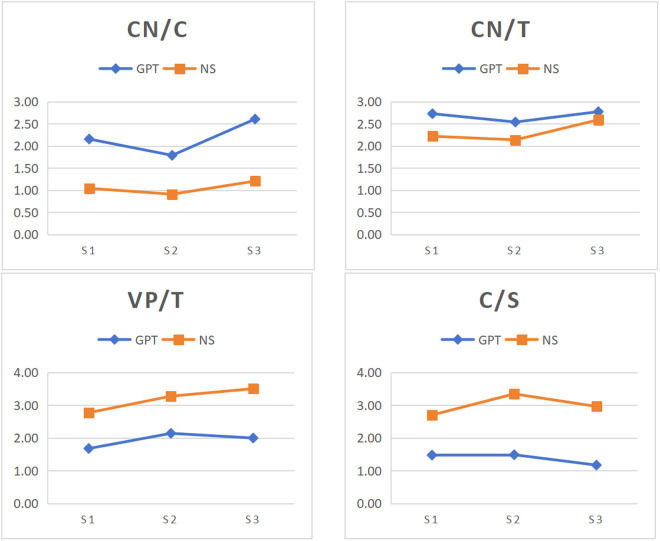
Variation of degree of phrasal sophistication and overall sentence complexity across rhetorical stages in GPT and NS. Note: S1, S2 and S3 represent Thesis, Argument and Conclusion. GPT and NS denote ChatGPT and native speakers.

According to the findings of stage-specific differences in syntactic complexity between ChatGPT and native speakers, the variation patterns across rhetorical stages were compared between ChatGPT-generated and native speakers-authored texts. Figure 1-4 illustrates the changes in syntactic complexity measure across rhetorical stages for ChatGPT and native speakers.

Fig 1 illustrates the means of three length of production unit measures in every stage for ChatGPT and native speakers. For MLC, MLS, and MLT, ChatGPT and native speakers demonstrate different variation patterns across rhetorical stages. In ChatGPT, all these three measures peaked in the conclusion stage and dropped to their minimum in the argument stage. Comparatively for native speakers, there were significant differences for MLC in the thesis and conclusion stages, with the conclusion stage being significantly higher. For MLS and MLT, both the argument and conclusion stages were significantly higher than the thesis stage.

Fig 2 illustrates the means of four amount of subordination measures in every stage for ChatGPT and native speakers. Both groups displayed similar overall variation patterns across rhetorical stages, but differences still emerged in each group. In ChatGPT, all four measures followed a consistent trend across stages, with the argument stage showing significantly higher values than the thesis stage. But in native speakers, C/T was highest in the argument stage.

Fig 3 illustrates the means of three amount of coordination measures in every stage for ChatGPT and native speakers. For CP/C and CP/T, the conclusion stage was the highest in both groups. In ChatGPT, the conclusion stage had significantly higher values than both the thesis and argument stages. Similarly, in native speakers, the conclusion stage exhibited significantly higher values than the argument stage, which, in succession, was higher than the thesis stage. For T/S, the two groups displayed contrasting trends. In ChatGPT, the conclusion stage was significantly higher than the argument stage, while native speakers were highest in the argument stage.

Fig 4 illustrates the means of three phrasal sophistication measures and one overall sentence complexity measure in every stage for ChatGPT and native speakers. In general, ChatGPT exhibited more significant between-rhetorical-stage differences compared to native speakers for these measures. Neither group showed significant difference across rhetorical stages for CN/T. For CN/C, contrasting to ChatGPT, native speakers exhibited no significant difference. For VP/T, both groups exhibited different patterns. In ChatGPT, the argument stage showed the highest value, whereas the thesis stage showed the lowest. In native speakers, the conclusion stage was the highest, with the thesis stage being the lowest. For C/S, both groups exhibited the highest values in the argument stage. However, the lowest value for ChatGPT was in the conclusion stage, whereas for native speakers, it was in the thesis stage.

## Discussion

This section discusses the key findings regarding the syntactic complexity and rhetorical stages in argumentative essays produced by ChatGPT and native speakers. It is organized into two subsections. The first examines syntactic complexity differences between ChatGPT and native writers within each rhetorical stage. The second compares the cross-stage variation patterns of syntactic complexity between the two groups.

### Differences between ChatGPT and native speakers by rhetorical stages

Syntactic complexity across different rhetorical stages produced by ChatGPT and native speakers was one of the focuses of the current study. The thesis and conclusion stage did not suggest significant differences between ChatGPT and native speakers in part of length of production units and subordination units. These findings suggest that ChatGPT’s writing performance in these specific measures and stages closely aligns with that of native speakers in argumentative essays, further highlighting ChatGPT’s capability of producing texts that approximate native-like quality in certain aspects of argumentative writing [19].

First, ChatGPT produced significantly longer T-units than native speakers in the thesis stage, which may be attributed to ChatGPT’s information density that aligns with the prompts of argumentative writing, which embeds more information within a single T-unit in the thesis stage. In contrast, native speakers convey their core message directly, emphasizing clarity and conciseness expected in thesis statements [30]. This difference is illustrated in Examples 1 and 2, both as opening sentences of the essay.

Ex. 1. In my opinion, smoking should be completely banned at all restaurants in the country due to health concerns, the impact on non-smokers, and the need for creating cleaner environments. (ChatGPT-thesis)
Ex. 2. I don’t think that smoking should be banned at all the restaurants in Japan. (native speakers-thesis)

And in the argument stage, native speakers elaborate on their arguments with more words within a single T-unit, approaching more clarified expressions of their intended meaning and making the arguments more persuasive. This difference is illustrated in Examples 3 and 4: Example 3 demonstrates how native speakers provide detailed evidence to support their claims, whereas Example 4 shows ChatGPT presenting a less detailed explanation.

Ex. 3. Next, I think smoking is disrespectful to the people who work at the restaurant. Even though the other customers may only have to endure the second hand smoke for one or 2 hours, the restaurants staff has to work for as many as 8 hours or more at a time, and so they will have to be around smoke much longer. (native speakers-argument)
Ex. 4. Finally, the presence of smoking areas within restaurants can also create discomfort for customers who do not smoke. The lingering smell of smoke can taint the dining experience and cause unease. (ChatGPT-argument)

Second, ChatGPT produced significantly longer clauses than native speakers across all rhetorical stages, while its MLS were notably shorter than native speakers in the argument and conclusion stages. This partially aligns with Zindela’s [36] study, which reported that ChatGPT-generated argumentative essays featured lower MLS compared to human writers. The reason is that human writers, as Zhou et al. [43] noted, often rely on longer sentences to highlight key information and convey complex ideas. Our finding indicates that ChatGPT tends to generate longer clauses within shorter sentences in the argument and conclusion stages. Although clauses help link ideas and convey complex expressions [44], shorter sentences are less effective than longer sentences in expressing complicated meanings [45], thereby limiting the depth and complexity of argumentation essential for persuasive argumentative writing.

Third, ChatGPT used more CN/C and CN/T in all or some stages, indicating a tendency to produce denser nominal structures. This partially aligns with Wang’s finding [46], which showed that a notable increase in CN/C in student essays revised by ChatGPT compared to the original versions. High-level writers tend to employ more complex phrases, with the frequent use of nominal phrases standing out as a distinctive feature in academic English writing [39], which means ChatGPT’s capacity to mimic advanced writing patterns by increasing complex nominals in its generated text, similar to that of proficient writers. In contrast, native speakers produced more VP/T than ChatGPT across all stages, indicating their more frequent use of verb phrases. For instance, Example 3 illustrates this with nine verb phrases (*is disrespectful to the people who work at the restaurant, work at the restaurant, may only have to endure the second hand, to endure the second hand, smoke for one or 2 hours, has to work for as many as 8 hours or more at a time, to work for as many as 8 hours or more at a time, will have to be around smoke much longer* and *to be around smoke much longer*). By using verb phrases, native speakers include richer semantic information, constructing layered and nuanced arguments with greater logical depth. This frequent use of verb phrases enhances the clarity and persuasiveness of their writing, making their arguments more comprehensive and compelling.

Finally, ChatGPT used more CP/C and CP/T, alongside significantly lower amount of subordination measures and C/S across all stages. This is in line with Tudino and Qin’s [21] findings, suggesting that ChatGPT relies more on simpler sentence structures with coordination phrases, rather than the hierarchically layered subordinate structures typically used by native speakers. Subordinate clauses, as a form of structural expansion, reduce the cognitive effort required from readers by explicitly specifying the semantic relationships between ideas, thereby enhancing the readability of the text [28,47]. Therefore, ChatGPT’s coordination-heavy approach may place a greater cognitive burden on readers. Example 5, a ChatGPT text of conclusion section, includes one coordination phrase (*ensuring comfort for all patrons, and promoting cleaner, healthier environments*), but lacks complex T-units or dependent clauses. In contrast, example 6, a typical native speaker’s text, includes one complex T-units (*I think that banning smoking in public places like restaurants is a great step in discouraging people from hurting themselves)*, one dependent clauses (*that banning smoking in public places like restaurants is a great step in discouraging people from hurting themselves*) but no coordination phrase. These examples highlight contrasting syntactic strategies in the conclusion stage: ChatGPT prioritizes clarity and simplicity through parallel structures, whereas native speakers employ subordinate structures to create more nuanced and reflective conclusions.

Ex. 5. In conclusion, the complete ban of smoking in all restaurants is essential for protecting public health, ensuring comfort for all patrons, and promoting cleaner, healthier environments. (ChatGPT-conclusion)
Ex. 6. I think that banning smoking in public places like restaurants is a great step in discouraging people from hurting themselves. (native speakers-conclusion)

However, native speakers produced more T/S than ChatGPT across all stages. A T-unit, defined as a production unit within a sentence that includes one main clause and any subordinate clauses directly or indirectly connected to it, serves as a key indicator of syntactic complexity [27]. As mentioned earlier, all measures related to subordination were consistently higher for native speakers across all stages. This suggests that native speakers incorporate both coordination and subordination structures in argumentative writing, often employing multiple T-units within a single sentence. In example 7, a native speaker’s argument text, the two sentences represent two T-units and complex T-units, with four dependent clauses (*that it is much more healthy for the other people in the restaurants if smoking is banned at the restaurants, if smoking is banned at the restaurants, as the secondhand smoke is much more dangerous than the smoke they are inhaling* and *they are inhaling*). This strategy enables the progressive development of arguments and a step-by-step articulation of viewpoints, thereby increasing the overall persuasiveness and coherence of argumentation.

Ex. 7. Next, I agree that it is much more healthy for the other people in the restaurants if smoking is banned at the restaurants. It is not right to have to breathe in the secondhand smoke of many smokers around you as the secondhand smoke is much more dangerous than the smoke they are inhaling. (native speakers-argument)

It is noteworthy that the two groups differed in variation. Higher standard deviations in native speaker texts reflect greater individual differences in syntactic complexity, likely due to diverse writing styles, rhetorical choices, and language proficiency. In contrast, the lower variation in ChatGPT outputs points to more uniform syntactic patterns. As Jiang and Hyland [15] observe, ChatGPT language tends to be more rigid and formulaic, lacking the flexibility and stylistic diversity characteristic of human writing. While such consistency ensures structural stability, it may constrain the range of syntactic choices essential for nuanced academic expression.

### Differences between ChatGPT and native speakers in cross-rhetorical stage variation pattern

Our study investigates ChatGPT’s and native speakers’ text syntactic complexity variation across rhetorical stages. We found that there is no variation in CN/T across all stages, the same for ChatGPT and for native speakers. This is an indication that ChatGPT can achieve a considerably comparable level of writing to native speakers in the use of complex nominal structures regardless of rhetorical stage.

In the thesis stage, ChatGPT used the fewest VP/T, while producing more CN/C compared to the argument stage, though still fewer than in the conclusion stage. Considered as the first stage, the thesis stage primarily focuses on presenting the writer’s stance or proposition, requiring clarity and conciseness rather than detailed elaboration [30,34]. This could explain the lowest VP/T in the thesis stage, as fewer verb phrases are needed for argument expansion at this stage. Furthermore, argumentative writing, as a frequent genre of academic writing, often features a condensed structure, incorporating phrasal modifiers within noun phrases and is much less explicit [28]. The intermediate use of CN/C indicates that complex nominal structures were employed to convey the core message precisely while avoiding overly intricate expressions. This balance between informativeness and readability is crucial for effectively establishing the argumentative foundation in the thesis stage.

In the argument stage, ChatGPT produced the shortest mean length of production units and used the fewest CN/C, while employing the highest amount of subordination and the most VP/T. This is possibly because, in the argument stage, writers are expected to express a stance clearly and substantiate this position with compelling evidence to effectively persuade the reader [48]. The greater use of subordination measures and VP/T suggest that ChatGPT can employ subordinate structures and verb phrases to connect and support multiple arguments, enabling a layered presentation of evidence that enhances persuasiveness. Meanwhile, the shortest mean length of production units and the fewest CN/C indicate ChatGPT’s preference for concise and straightforward expressions. While this approach enhances clarity, it may limit information density and depth, particularly in the argument stage, where more detailed evidence is often required to build a robust and convincing argument [34].

The conclusion stage witnessed the highest CN/C and T/S, but the lowest C/S. This suggests that ChatGPT employs more T-units, clauses and denser nominal structures to list the core arguments effectively, aligning with the summarizing function of the conclusion stage. Meanwhile, the lowest C/S reflects a simplification of syntactic complexity, likely aimed at enhancing readability and ensuring the summary remains concise and direct.

The overall syntactic complexity variation pattern across rhetorical stages for ChatGPT reflects its ability to adapt syntactic strategies to the communicative functions of different rhetorical stages in argumentative essays. It suggests that ChatGPT’s syntactic complexity varies systematically across stages, aligning with the functional demands of each rhetorical section.

Regarding native speakers, the pattern of syntactic complexity variation across part of genres differed from that of ChatGPT in some measures. In addition to CN/T, as previously mentioned, no significant differences were observed across rhetorical stages in CT/T, DC/C, DC/T, and CN/C. This suggests that native speakers consistently employ complex nominals and subordinate structures across rhetorical stages to achieve overall writing objectives. Such stability highlights their adaptability to varying rhetorical contexts without depending on a single syntactic strategy, reflecting their advanced proficiency in argumentative writing [23].

In the thesis stage, native speakers produced the shortest MLS and MLT, as well as the fewest VP/T and C/S, suggesting the tendency to use shorter sentences and T-units, with reduced reliance on verb phrases and clauses. This reflects a deliberate strategy in the thesis stage, which is to introduce the central argument clearly and concisely, ensuring that the reader quickly grasps the core focus of the essay [30,34]. Moreover, the use of shorter sentences and fewer clauses usually minimizes the cognitive load on readers [49], allowing grasping the proposition more efficiently and focusing on the central message.

In the argument stage, native speakers used more C/T and T/S, indicating that native speakers tend to construct sentences with more clauses and longer T-units to develop their arguments. This reflects the functional demands of the argument stage, where claims must be elaborated and supported with detailed reasoning and evidence [44]. The use of more C/T enables sentences to embed multiple logical relationships, such as causality, contrast, or condition, enriching the argument’s depth and precision. Similarly, longer T-units allow the integration of layered reasoning and supporting evidence within a single sentence, reducing fragmentation and ensuring logical coherence. By employing these syntactic strategies, native speakers enhance both the persuasiveness and cohesiveness of their arguments, effectively meeting the rhetorical demands of the argument stage.

In the conclusion stage, native speakers produced the longest clauses and used the most VP/T. This is consistent with the functional demands of the conclusion stage, which requires both a comprehensive synthesis of key arguments and a clear reinforcement of the central claim [34]. The use of longest clauses enables the integration of multi-layered information, while verb phrases facilitate logical connections between summarized ideas. This approach enhances the depth and clarity of the summary, reflecting native speakers’ ability to balance syntactic complexity with readability in argumentative writing.

The overall syntactic complexity variation pattern across rhetorical stages for native speakers is less variable than that for ChatGPT, indicating that native speakers show greater consistency in choosing syntactic structures to fulfill the communicative functions across various stages. In the thesis stage, native speakers employ shorter sentences, T-units and fewer verb phrases, emphasizing clarity and conciseness to directly present their central argument. In the argument stage, they effectively utilize subordinate structures and achieve high T/S values, enabling them to progressively elaborate on their claims and integrate multi-layered information within single sentences. In the conclusion stage, native speakers rely on longer clauses, a higher density of verb phrases to summarize key arguments and maintain clarity and organization. Their ability to combine denser information units with various syntactic strategies highlights their advanced proficiency in constructing nuanced, purpose-driven academic texts.

## Conclusion

This study compared the syntactic complexity in ChatGPT’s and native speakers’ argumentative essays, especially from the perspective from rhetorical stages. The results indicated that while both groups showed similarities in part of syntactic complexity measures, some notable differences were observed. ChatGPT demonstrated more obvious favor toward using coordination and shorter sentence structures, particularly in the argument stage, while native speakers employed a greater variety of syntactic strategies, including more extensive subordination, which allowed for more nuanced argumentation and deeper logical relationships. Moreover, ChatGPT exhibited lower variation than native speakers in many syntactic complexity measures, suggesting a more rigid and formulaic language style [15].

This study holds significant implications. ChatGPT’s ability to produce well-structured argumentative texts demonstrates its potential to assist writing. For L2 learners, ChatGPT can serve as a valuable writing assistance tool, especially for enhancing syntactic complexity and adapting to rhetorical stages in argumentative essays. Its ability to produce coherent outputs across rhetorical stages contributes to the development of learners’ genre awareness. For educators, this study highlights the potential of using ChatGPT as a supplementary resource which can be integrated into the language classrooms. Educators should also guide students in strengthening their syntactic strategies and ensuring the depth of their rhetorical structures, moving beyond ChatGPT’s strengths and addressing its limitations in more nuanced writing tasks.

This study also has several limitations. First, it concentrated on syntactic complexity without considering other aspects such as coherence, lexical choice and writing style. Second, the study was based on a limited sample size of 146 essays. Third, the study focused solely on ChatGPT and argumentative essays, ignoring other large language models such as DeepSeek or Claude, as well as other genres like narrative or descriptive essays. Future studies should encompass more linguistic features, large language models and writing genres, as well as a larger sample size to provide a more comprehensive view of AI’s and human writers’ performance in English writing.

## Supporting information

S1 File (ZIP)
